# Assessment of hair and cashmere properties and their genetic background of several goat breeds in Southwest China

**DOI:** 10.1038/s41598-022-14441-1

**Published:** 2022-07-01

**Authors:** Ahmed A. Saleh, Amr M. A. Rashad, Nada. N. A. M. Hassanine, Mahmoud A. Sharaby, Yongju Zhao

**Affiliations:** 1 Animal and Fish Production Department, Faculty of Agriculture (Alshatby), Alexandria University, Alexandria City, 11865 Egypt; 2 College of Animal Science and Technology, Southwest University, Chongqing Key Laboratory of Forage and Herbivore, Chongqing Engineering Research Center for Herbivores Resource Protection and Utilization, Chongqing, 400715 People’s Republic of China

**Keywords:** Genetics, Physiology, Zoology

## Abstract

The aim of this study was to determine the properties and quality characteristics of hair and cashmere fibres of three goat breeds raised in Southwest China, namely; Dazu black goat (DBG, n = 203; ♂99, ♀104), Inner Mongolia cashmere goat (IMCG, n = 65; 21♂, 44♀) and their first cross (F_1_, n = 79; 39♂, 40♀). Totals of 5219, 2130 and 2981 fibre samples, from the three breeds respectively, were taken prior to shearing at 32.5 ± 01.25 months of age from four body sites; shoulder, side-portion, abdomen and leg. Breed effect was significant (*P* < 0.01) for most hair and cashmere properties. IMCG and F_1_ hair lengths were longer (*P* < 0.001) with less variable lengths than DBG. Shoulder hair diameters of the three breeds were not different (*P* > 0.05) but biggest of the side-portion and abdomen sites of DBG were bigger (*P* > 0.01), however, the biggest (*P* < 0.001) hair diameter was recorded for the leg site of F_1_ and the smallest (*P* > 0.01) for IMCG. IMCG recorded the longest value for cashmere lengths followed by DBG, while F_1_ recorded the lowest (*P* = 0.001), whilst F_1_ recorded the biggest (*P* = 0.001) diameter whereas no differences existed between parents' breeds. The cortical cell lengths of IMCG and DBG were 94.57 and 86.85 μm without significant difference detected between breeds. Differences between hair length and diameter for body sites of the studied goat breeds were significant (*P* < 0.01) but between whiteness, cashmere diameter and diameter of cortical cells were not. Sex had no significant effect on all hair/cashmere properties. Quality characteristics of cashmere fibres from IMCG and F_1_ were better (*P* < 0.001) than from DBG. Leg hair diameter, curl recovery rate and cashmere diameter were superior in the crossbred F_1_ compared to pure breed parents, and DBG was superior to IMCG for fibre elasticity and intensity traits. *FGF-5* gene was detected as a candidate gene for hair and cashmere traits in IMCG breed. Whilst, *KIT* gene was found to be associated with coat colour in the studied breeds. Extra investigations to examine more cashmere goat breeds and crosses are needed to discover genetic variability in cashmere production locally and worldwide.

## Introduction

Throughout many domestication events, several goat breeds have stepped into the luxury fibre production field^1,2^. Hair/fibre is an important product from goats kept in some of the most marginal inhospitable agriculture areas of the world. There are three significant fibre types produced from goats; cashmere, mohair, and hair. The fourth is cashgora, a hybrid type, with intermediate characteristics between cashmere and mohair. The important physiological difference between mohair and cashmere production lies in the response of fibre growth to changing levels of nutrition. The yield and diameter of cashmere fibres are highly unresponsive to changing nutritional status. Also, cashmere growth is remarkably insensitive to the nutritional influence, and may successfully be produced in harsh continental climates^3–5^.

The cashmere, is a fibre collected from cashmere, pashmina and several other goat breeds. It is stronger, finer, softer, lighter, and approximately three times more insulating than sheep wool^6^. The hair/fibre of the cashmere goats consists of two types; (1) fibre covering the goat body, known as guard hair or outer coating coarse fibre and (2) down fibre or undercoat cashmere, soft and fine fibre protects goats from cold^8^. In south Asia, cashmere is called "pashmina" a Persian word that means "fine wool"^9^. The guard hair, could be short or long, but straight, coarse and distinguishable from the undercoat cashmere fibres. Cashmere fine fibres must be separated from guard hair before being sold or processed. In the industry, this step is known as ''Dehairing'', and should take place before fibre dyeing and converting into textile yarn, fabrics then clothes^8^.

China, one of the largest cashmere producing countries^10^, has an estimated population of more than 1,230,000 heads of goats that produce around 10,000 tons of cashmere presenting 40% of world production^11^.

Goat hair/cashmere is used for the manufacture of coarse cloth, tent fabric and rope^7^, while cashmere wool is one of the most expensive fibres commercially produced. Cashmere is harvested once a year, yielding about 260 g of fibre/head^9^.

Cashmere goats are economically important livestock sector distributed mainly in remote semi-arid areas of Asia especially in north China, Mongolia and recently in southwest China^12,13^. Although cashmere breeds raised around and in the Tibetan mountains have extremely unique soft fibres^14^, cashmere could be produced from other breeds/strains, in other locations around the world. For example; in Asia and Europe, cashmere goats are geographically found between 50°–120° east longitude and 35°–55° north latitude. These breeds are adapted to cold regions and raised in areas at more than 1000 m above sea level^7^. In general, goat breeds in China are distributed in five eco-economical regions; Northern pasture, Qinghai-Tibetan high land pasture, Agriculture-Animal husbandry integrated region and Northern and Southern Agricultural regions^7^.

Several cashmere breeds have been moved from different regions of China to Southwest China including Inner Mongolia cashmere goat (IMCG) to be raised with the local Dazu black goat (DBG) breed which is native of Chongqing. It is characterized by high adaptability and sound reproductive performance, therefore, is used extensively for breeding purposes^15^.

In fact, Chinese cashmere as being the best fibres is utilized in making luxury tricot^16^. Several investigations confirmed that; the value of Chinese cashmere fibre is related primarily to its small fibre diameter and is influenced by secondary characteristics, such as lustre, colour, elasticity, intensity, whiteness and fineness, in addition to yield and proportion of down fibres in total fleece^17^.

Also, the coat colour plays a necessary role in the adaptation, dodge predators in harsh environments and indirect effects on the productive traits^18,19^, it would be wonderful to investigate trait like that and its genetic background, besides the properties and quality characteristics of hair and cashmere fibres in the goats. Many genes like; *FGF-5*^20^, *KIT*, *IGFBP-7* and *PLOD3*^21^ have been reported to be correlated with different hair/cashmere traits, such as; type, appearance, length, diameter, coat colour, etc. in several goat breeds.

Few investigations are available on the differentiation of hair/cashmere properties and quality characteristics over the body sites of cashmere goats and their crosses and yet, research on the down-fibre characteristics of native goat breeds in China are still limited. To the best of our knowledge, no comprehensive studies have examined differences in hair and cashmere characteristics between different genotypes/breeds or their association with genetic reasonings.

So, this study is a part of a comprehensive investigation for characterization of several goat breeds raised in Southwest China. Evaluation of morphological traits, physiological variables and fibre properties, involving the use of whole-genome sequencing (WGS) and genome-wide association studies (GWAS) are being undertaken with a special focus on the coat colour of IMCG, DBG and their first cross (F_1_). The impact of crossing, and *FGF-5* and *KIT* (rs647214940 site) genes on fibre properties and coat colour, respectively, were appraised.

## Results

Genotype/breed effect was highly significant (*P* < 0.001) for all hair and cashmere properties and quality characteristics, except; curl degree, elongation at break cortical cell dimensions (length and diameter) which was different at (*P* = 0.033), (*P* = 0.032), (*P* = 0.148) and (*P* = 0.559), respectively, but no effects were detected for sex or interaction between breed and sex (*P* > 0.05) (Supplementary File 1, Tables S2: S8). Sample site also affected hair and cashmere length and diameter (*P* < 0.001) of IMCG and F_1_ (Tables 1, 2, 3, 4).

**Table 1:** Hair length and diameter of IMCG^1^, DBG^2^ and their first cross F_1_^3^ (mean ± SEM).

Hair traits	Breed			SEM^4^	*P*-value
	IMCG^1^	DBG^2^	F_1_^3^		
**Hair length (cm)**					
Shoulder	18.380^a^	4.032^c^	11.406^b^	0.826	< 0.001
Side portion	11.975^a^	4.015^c^	6.797^b^	0.542	< 0.001
Abdomen	9.955^a^	4.120^c^	6.350^b^	0.440	< 0.001
Leg	15.630^a^	4.400^c^	11.019^b^	0.735	< 0.001
Overall mean	14.901^a^	4.049^c^	8.901^b^	0.636	< 0.001
**Hair diameter (μm)**					
Shoulder	88.910	91.044	90.495	2.172	0.906
Side portion	84.182^b^	99.765^a^	83.270^b^	2.264	0.001
Abdomen	85.553^a^	89.213^a^	68.700^b^	1.396	< 0.001
Leg	79.773^c^	89.678^b^	103.061^a^	1.921	< 0.001
Overall mean	84.601^c^	92.424^a^	86.381^b^	1.935	0.004

^1^IMCG; Mongolia Cashmere goat, ^2^DBG; Dazu black goat, ^3^F_1_; IMCG DBG cross. ^4^SEM; the standard error of the mean.

^a–c^Means with different superscript in the same row are differ (*P* < 0.05).

**Table 2:** Cashmere fibre length and diameter of IMCG^1^, DBG^2^ and their first cross F_1_^3^ (mean ± SEM).

Cashmere traits	Breeds			SEM^4^	*P*-value
	IMCG^1^	DBG^2^	F_1_^3^		
**Cashmere length (cm)**					
Shoulder	5.012^a^	3.679^b^	2.411^c^	0.095	< 0.001
Side portion	4.243^a^	3.061^b^	2.223^c^	0.105	0.001
Abdomen	4.512^a^	2.336^b^	2.209^b^	0.123	0.001
Leg	5.003^a^	2.407^b^	2.347^b^	0.088	< 0.001
Overall mean	4.549^a^	3.921^b^	2.352^c^	0.102	0.001
**Cashmere diameter (μm)**					
Shoulder	14.929^b^	15.147^b^	16.904^a^	0.117	0.002
Side portion	13.417^b^	15.187^a^	15.409^a^	0.118	< 0.001
Abdomen	13.327^b^	14.969^a^	15.348^a^	0.162	0.004
Leg	14.468^b^	15.024^b^	16.913^a^	0.126	< 0.001
Overall mean	15.760^b^	15.922^b^	16.271^a^	0.131	0.001

^1^*IMCG* Mongolia Cashmere goat, ^2^*DBG* Dazu black goat, ^3^*F*_*1*_; IMCG*x*DBG cross. ^4^*SEM* the standard error of the mean.

^a–c^Means with different superscript in the same row are differ (*P* < 0.05).

**Table 3:** Quality traits of cashmere fibre of IMCG^1^, DBG^2^ and their first cross F_1_^3^ (mean ± SEM).

Quality traits	Breeds			SEM^4^	*P*-value
Cashmere length (cm)	IMCG^1^	DBG^2^	F_1_^3^		
Natural length	4.549^a^	4.049^b^	2.352^c^	0.076	0.001
Straight length	5.896^a^	4.869^b^	3.916^c^	0.088	< 0.001
**Cashmere elasticity**					
Curl degree (J)	2.862^b^	3.574^a^	2.890^b^	0.172	0.033
Curl recovery rate (JW/%)	74.49^b^	77.121^a^	78.071^a^	0.642	0.007
Curl modulus (JD/%)	2.138^b^	2.298^a^	1.912^c^	0.074	0.002
**Cashmere intensity**					
Elongation at break (mm)	4.955^a^	2.956^b^	2.593^b^	1.047	0.032
Breaking strength (cN)	12.266^c^	38.287^a^	27.910^b^	0.899	< 0.001
Work (cN*mm)	40.332^c^	59.536^a^	46.983^b^	2.675	< 0.001
Strength (cN/dT)	15.641^c^	40.071^a^	33.990^b^	0.932	< 0.001
EYS1.5 (mm)	4.019^a^	2.215^b^	1.990^b^	0.126	< 0.001
Yield point (cN)	11.519^c^	32.555^a^	28.982^b^	0.834	< 0.001
Elongation (%)	49.562^a^	25.347^c^	27.022^b^	1.004	< 0.001
**Cashmere whiteness**					
Shoulder	35.098^a^	27.514^c^	31.173^b^	0.801	0.008
Side portion	35.519^a^	27.324^c^	32.175^b^	1.210	0.009
Abdomen	35.506^a^	26.546^c^	31.050^b^	1.126	0.045
Leg	33.781^a^	26.534^c^	30.750^b^	0.935	0.028
Overall mean	34.988^a^	26.980^c^	31.289^b^	1.018	0.019

^1^*IMCG* Mongolia Cashmere goat, ^2^*DBG* Dazu black goat, ^3^*F*_*1*_; IMCG*x*DBG cross. ^4^*SEM* the standard error of the mean.

^a–c^Means with different superscript in the same row are differ (*P* < 0.05).

**Table 4:** Cortical cell length and diameter in IMCG^1^ and DBG^2^ parents goat breeds (mean ± SEM).

Cortical cells	Breeds		SEM^3^	*P*-value
	IMCG^1^	DBG^2^		
**Length (μm)**				
Shoulder	91.565	86.908	1.980	0.269
Side portion	93.933	86.788	2.658	0.198
Abdomen	97.150^a^	86.828^b^	2.719	0.045
Leg	94.215^a^	86.840^b^	2.156	0.083
Overall mean	94.575	86.854	2.378	0.148
**Diameter (μm)**				
Shoulder	5.588	5.183	0.326	0.576
Side portion	5.928	5.473	0.349	0.556
Abdomen	6.870	5.413	0.501	0.553
Leg	5.860	5.355	0.383	0.551
Overall mean	5.866	5.355	0.389	0.559

^1^*IMCG* Mongolia Cashmere goat, ^2^*DBG* Dazu black goat, ^3^*SEM* the standard error of the mean.

^a–c^Means with different superscript in the same row are differ (*P* < 0.05).

### Fibre length and diameter

#### The guard hair

**Figure 1:**
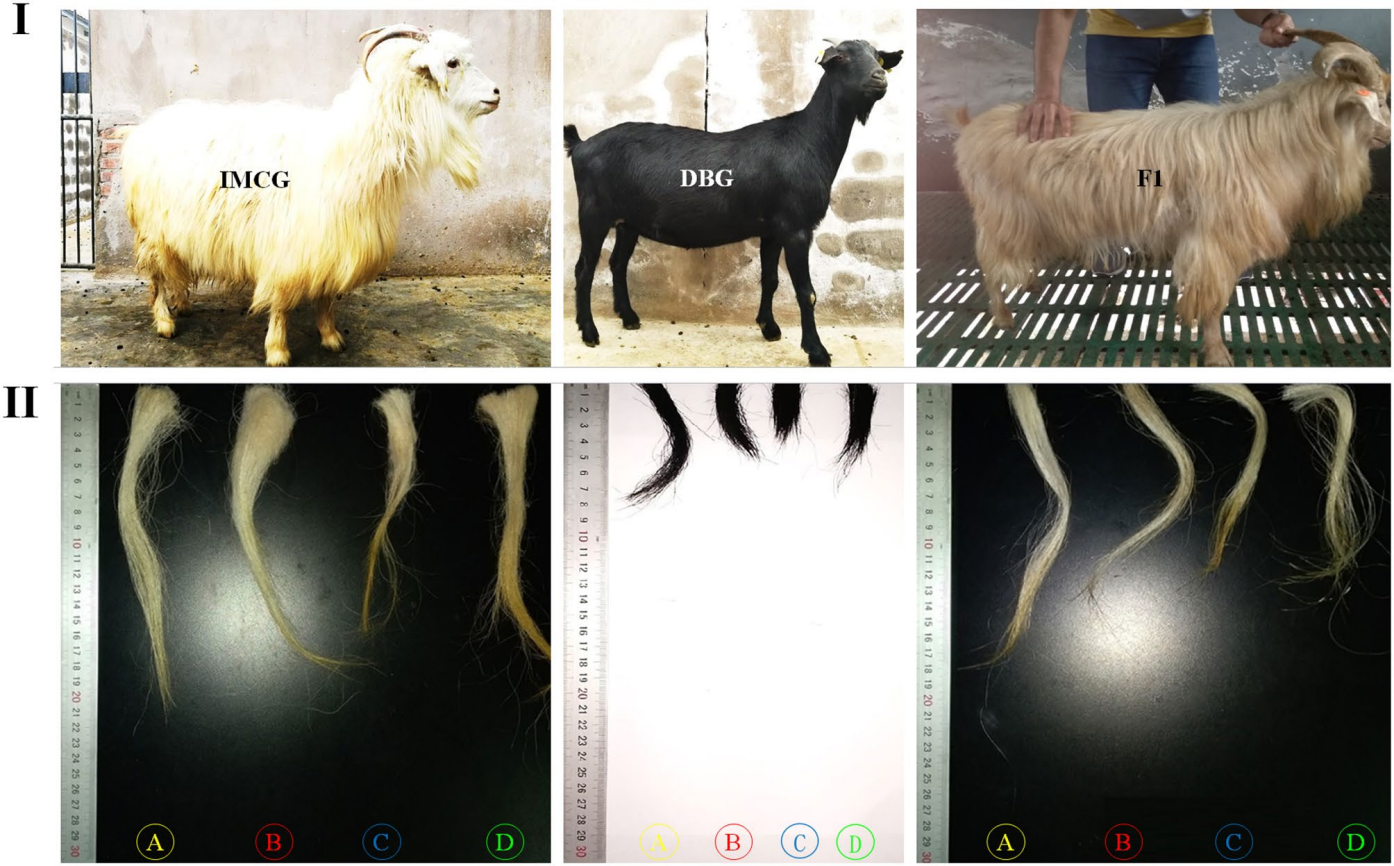
(**I**) The coat colour (white, black and off white to light-brown) and (**II**) Hair length of studied goat breeds; Inner Mongolia Cashmere goat (IMCG), Dazu black goat (DBG) and IMCG *×* DBG (F_1_), respectively. (A) shoulder, (B) side-portion, (C) abdomen and (D) leg sites.

There were highly significant differences (*P* < 0.001) between means of hair length of different goat breeds, IMCG produced the longest (*P* = 0.001) hair followed by F_1_ (*P* < 0.01) concomitantly with less variability than in DBG which recorded the lowest hair length. The same trend was found on hair length of all body sites (*P* < 0.001). Shoulder and leg hair lengths were longer than of abdomen and side-portion in IMCG and F_1_, comparing to DBG breed (*P* < 0.001) (Table 1 and Fig. 1).

**Figure 2:**
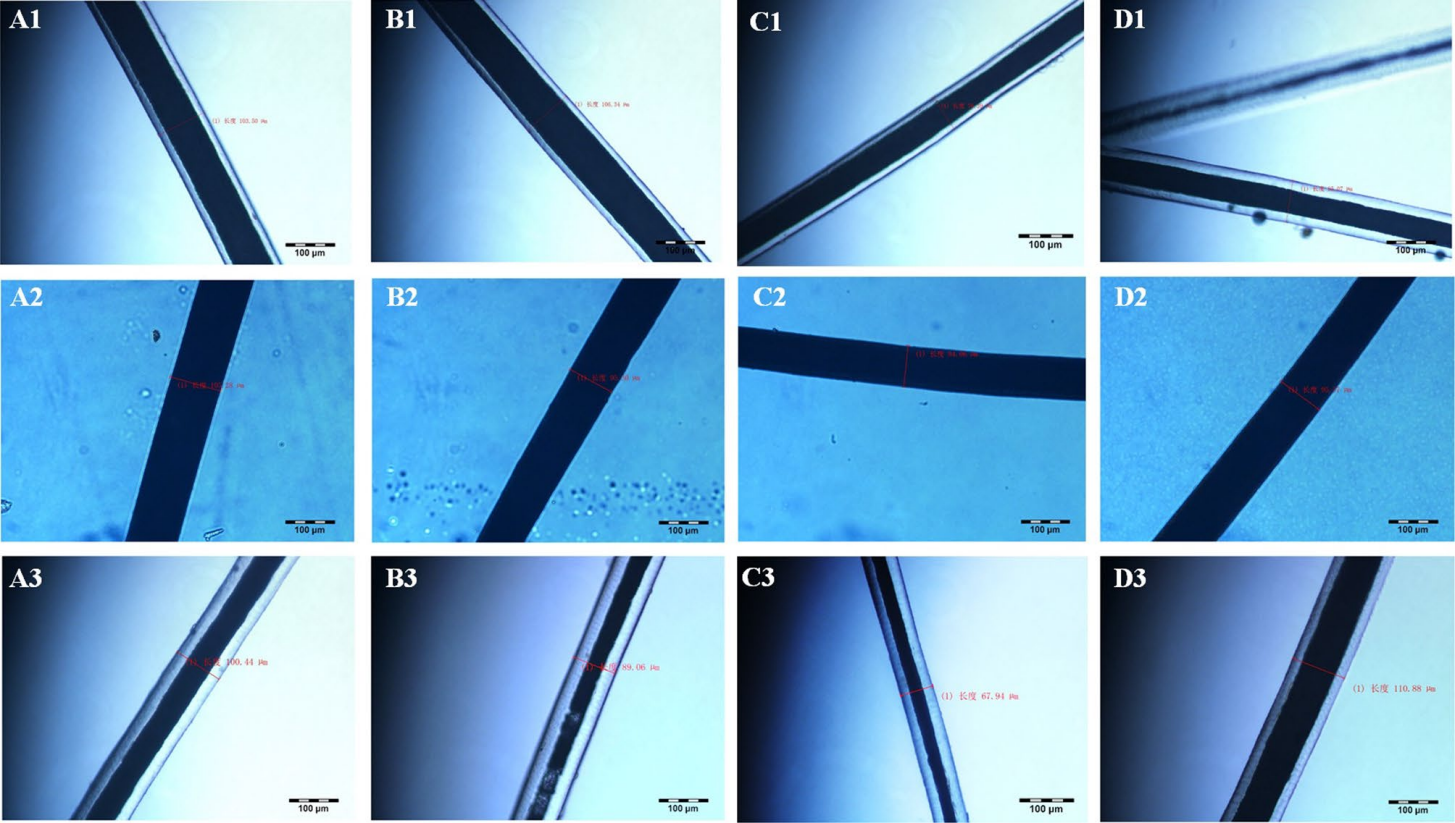
The diameter of hair of (**1**) Inner Mongolia cashmere goat (IMCG), (**2**) Dazu black goat (DBG) and (**3**) IMCG*x*DBG (F_1_) for (**A**) shoulder, (**B**) side-portion, (**C**) abdomen and (**D**) leg sites.

Also, significant differences (*P* = 0.004) were found between means of hair diameter of all studied breeds, but for shoulder site, no significant differences (*P* > 0.05) were observed. The highest (*P* < 0.001) value for leg hair diameter was recorded for F_1_, but the lowest was for IMCG (*P* < 0.01). For the side-portion site, the highest (*P* < 0.001) hair diameters were obtained on DBG and the other two breeds had similar diameters (*P* > 0.05). For abdomen hair, parents IMCG and DBG had similarly high diameters (*P* < 0.05) while F_1_ had the smallest (*P* < 0.001), (Fig. 2).

#### The down fibre cashmere

**Figure 3:**
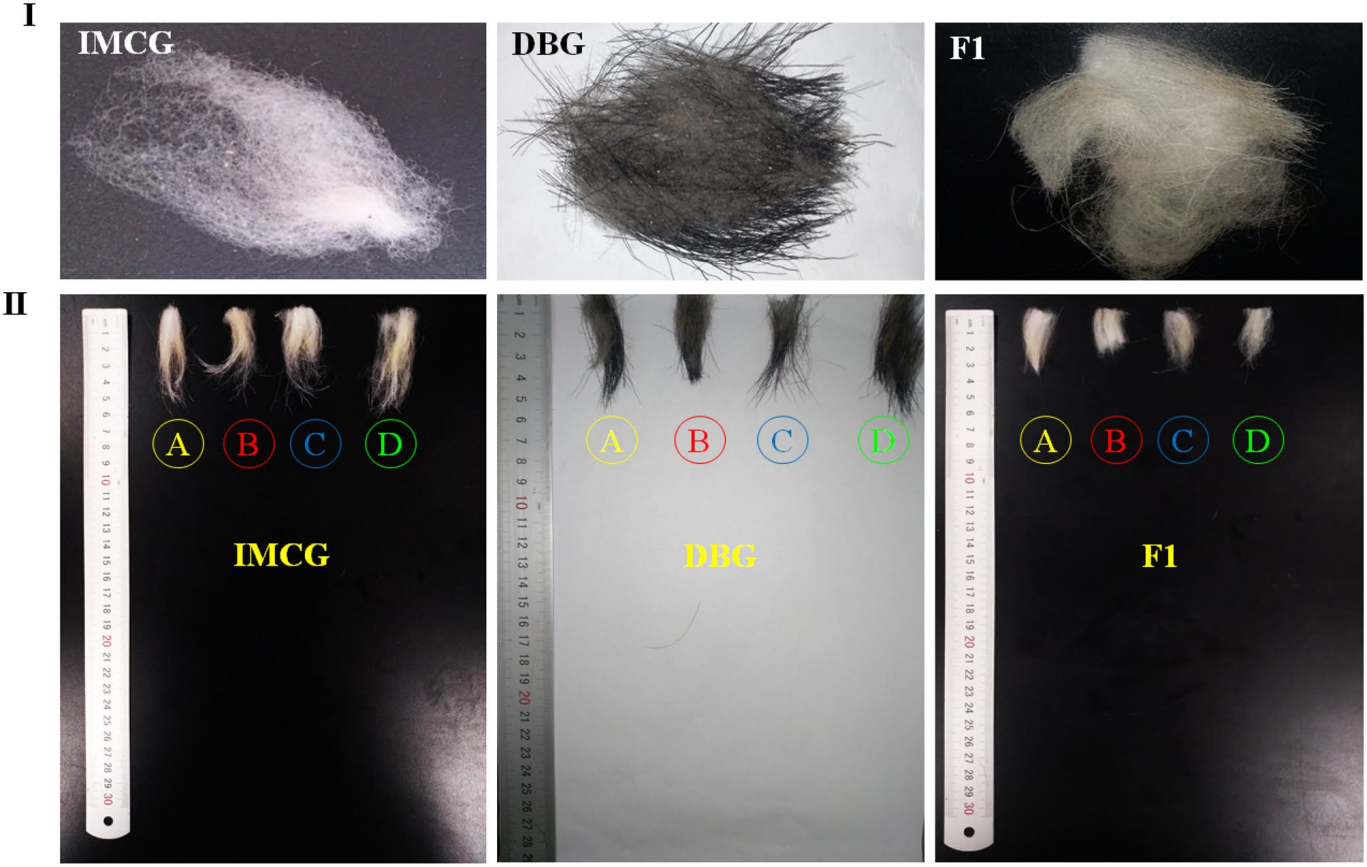
(**I**) Original cashmere samples and (**II**) Cashmere length of studied goat breeds; Inner Mongolia Cashmere goat (IMCG), Dazu black goat (DBG) and IMCG*x*DBG (F_1_). (A) shoulder, (B) side-portion, (C) abdomen and (D) leg sites.

There were highly significant differences (*P* = 0.001) between means of cashmere length of different goat breeds, IMCG produced the longest (*P* = 0.001) cashmere length followed by DBG concomitantly with less variability than in F_1_ which recorded the lowest cashmere length. Shoulder and leg cashmere lengths were longer than of abdomen and side-portion in all studied breed (*P* < 0.05) (Table 2 and Fig. 3).

**Figure 4:**
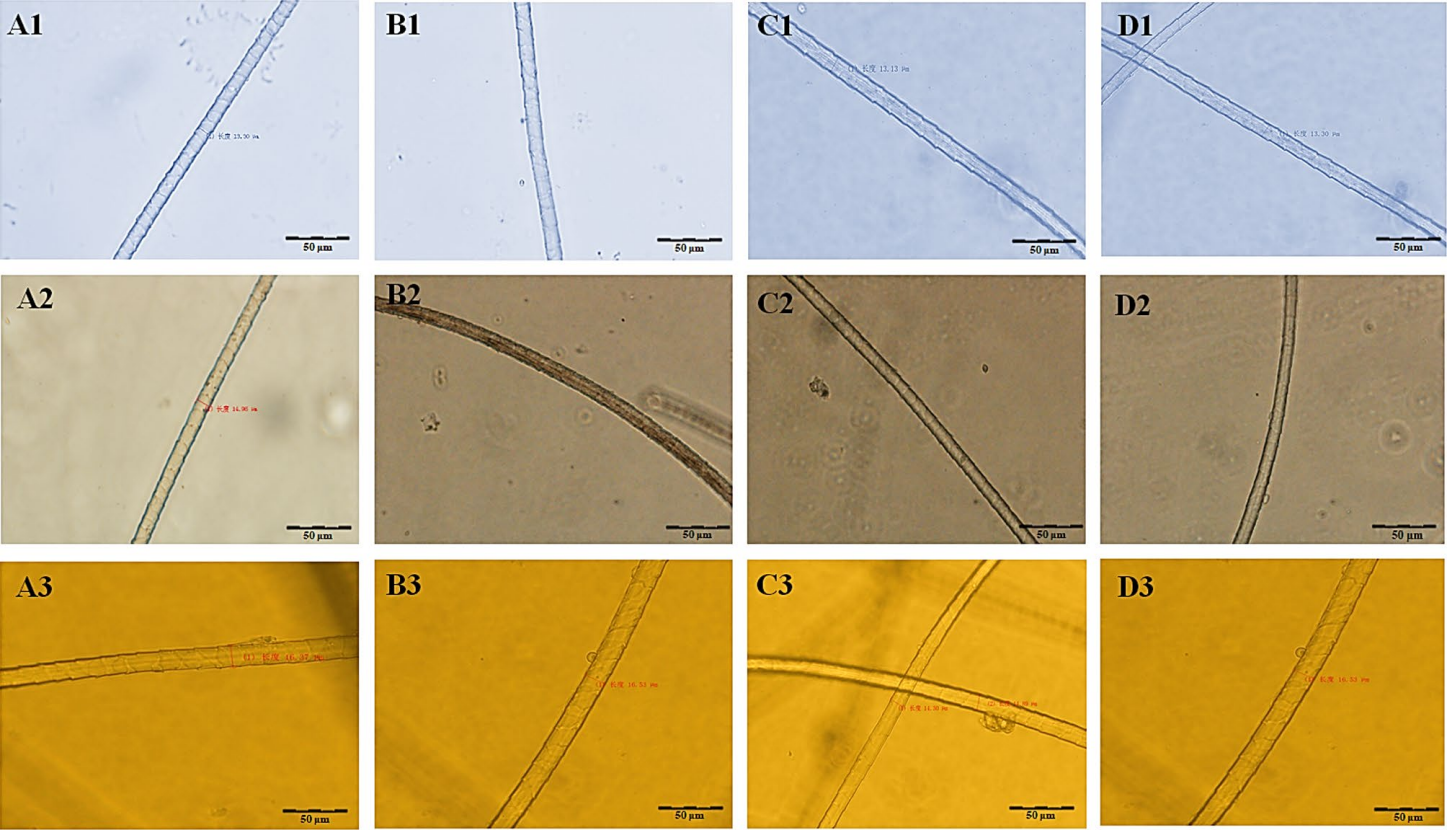
The diameter of cashmere of (**1**) Inner Mongolia cashmere goat (IMCG), (**2**) Dazu black goat (DBG) and (**3**) IMCG*x*DBG (F_1_) for (A) shoulder, (B) side-portion, (C) abdomen and (D) leg sites.

The average diameter of cashmere ranged between 15.76 and 16.27 μm for IMCG and F_1_. The difference between IMCG and DBG was not significant (*P* > 0.05), while F_1_ was significantly higher than both parents breeds (*P* < 0.01) though closer to DBG. Moreover, there are significant differences in cashmere diameter were detected among various body sites within each breed (Table 2 and Fig. 4).

#### Cashmere fibre quality traits

Quality traits of cashmere fibre of different goat breeds are shown in (Table 3). Significant differences (*P* < 0.001) were obtained between breeds for most quality traits of the studied breeds, while no differences (*P* > 0.05) were found between different body sites and both sexes within each breed (Supplementary File 1, Tables S5: S11).

Means of cashmere natural length and straight length of IMCG breed were higher (*P* ≤ 0.001) than those of DBG and F_1_, whilst F_1_ recorded the lowest means. All cashmere fibre elasticity variables were different (*P* < 0.03) among breeds. As for curl degree, the highest value was observed on DBG, followed by F_1_ and IMCG which were not significantly different. The highest curl recovery rate was recorded for F_1_ followed by DBG then IMCG but no significant differences were found between F_1_ and DBG. With regard to curl modulus, differences among all breeds were significant (*P* = 0.002), with the lowest value obtained for F_1_ and the highest for DBG.

All cashmere intensity traits were significantly different (*P* < 0.001) among breeds except elongation at break which was different at (*P* = 0.032). IMCG recorded the highest elongation at break (*P* = 0.032), elongation % and EYS1.5 (*P* < 0.001). While DBG recorded the highest values for intensity traits of breaking strength, work, strength and yield point (*P* < 0.001). For all cashmere intensity traits, F_1_ won intermediate positions between the values of both parents breeds. As for cashmere whiteness, the differentiation indicated significant (*P* = 0.019) variability among breeds, while no significant differences were found between body sites within each breed. The highest degree of whiteness was observed on IMCG, followed by F_1_, while the lowest degree was obtained on DBG.

#### Cortical cells dimensions

**Figure 5:**
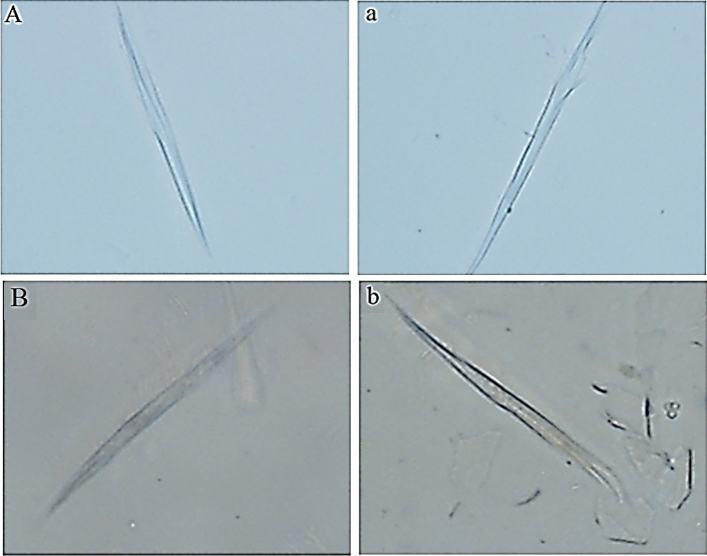
The cortical cells at (400x) magnification for (**A**) Inner Mongolia Cashmere goat, (**B**) Dazu black goat. The stromal cells at (400×) magnification for (**a**) Inner Mongolia Cashmere goat, (**b**) Dazu Black goat.

The mean length of the cortical cells of fibres from different body sites were slightly different (*P* = 0.148) between the two parents breeds (94.57 *vs* 86.85 μm), whilst for diameters (5.86 *vs* 5.35 μm) were not significant. However, the abdomen site of IMCG showed the highest cortical cell length (*P* = 0.045) and diameter (*P* = 0.543) among all body sites (Table 4). Figure 5 shows the cortical and stromal cells for both parents breeds.

#### Coat colour

**Figure 6:**
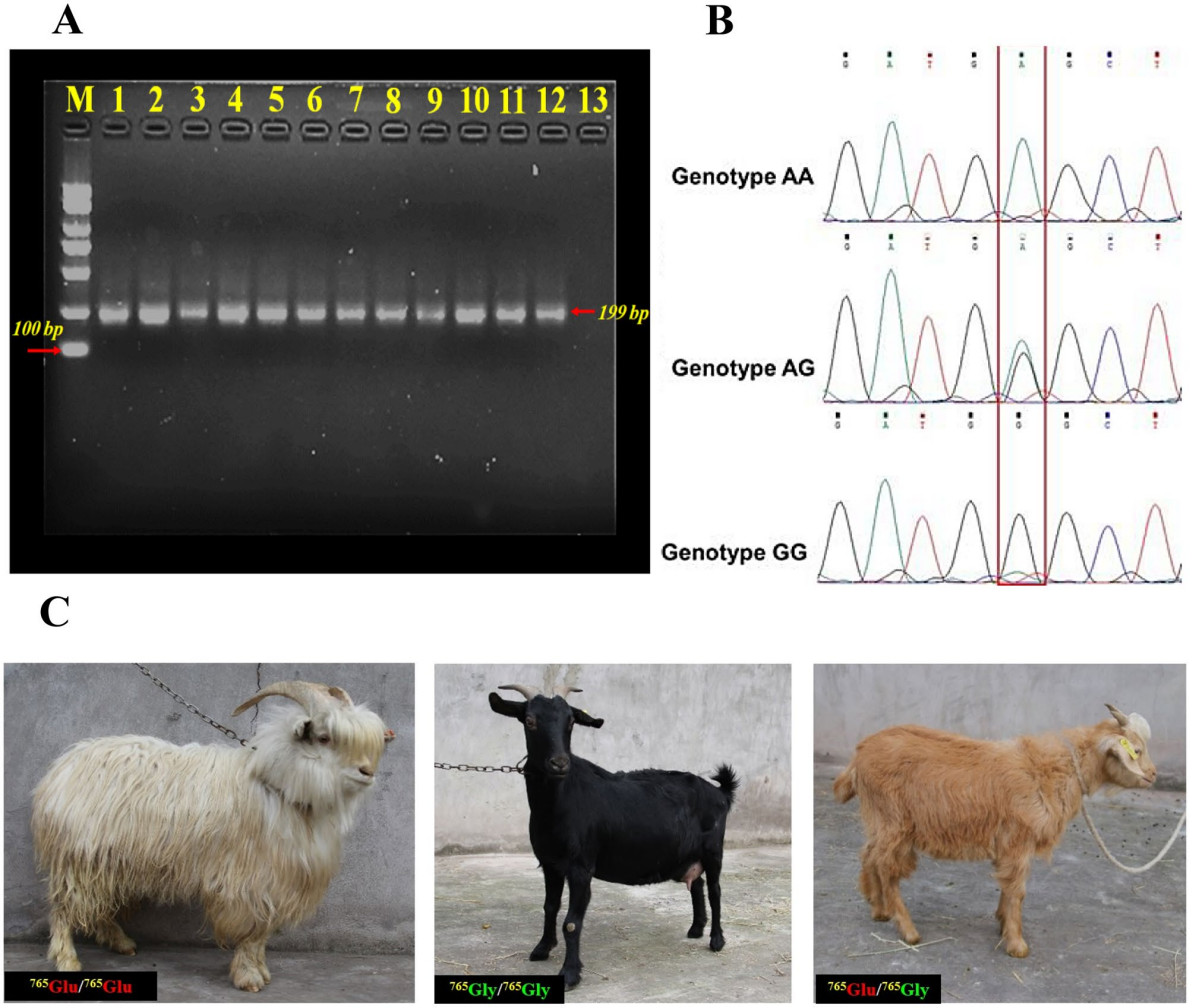
(**A**) PCR amplification (electrophoretogram) of *Kit* gene (rs647214940 site), Lanes: 1 to 4 for Inner Mongolia cashmere (IMCG), lanes: 5 to 8 for Dazu black goat (DBG), and lanes: 9 to 12 for IMCG*x*DBG cross (F_1_), M: 100 bp DNA ladder. (**B**) Sanger sequencing result for *Kit* gene (rs647214940 site) and (**C**) The non-synonymous mutation g. 68354798G>A (rs647214940 site) led to ^765^glutamic (Glu)/^765^glycine (Gly) (off white to light brown coat colour) in first cross (F_1_), while was ^765^Glu/^765^Glu (white coat colour) in Inner Mongolia Cashmere goat (IMCG), and was ^765^Gly/^765^Gly (black coat colour) in Dazu Black goat (DBG).

**Figure 7:**
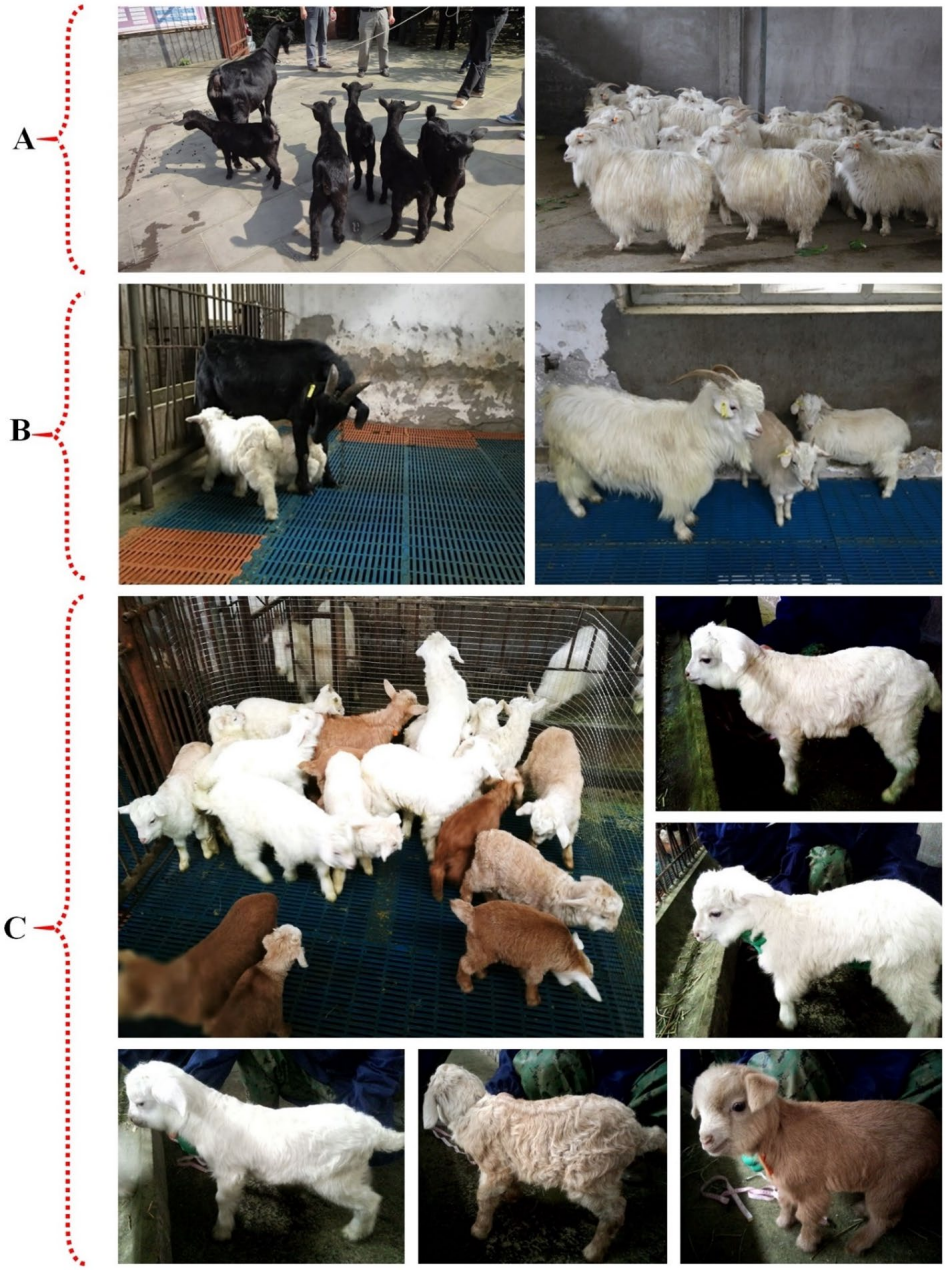
(**A**) The both pure breeds; Inner Mongolia Cashmere goat (IMCG) and Dazu Black goat (DBG), and their progeny (inbreeding). (**B**,**C**) The results of crossing between IMCG and DBG, and the light coat colour (off white to light-brown) for all the progeny (F_1_).

Three different colours were found in the studied goats; white, black and off white to light-brown for IMCG, DBG and F_1_, respectively (Figs. 6C and 7C, and Table 5). The coat colour of F_1_ was very close to the IMCG parent.

#### Hair/cashmere properties and their association with genetic variability

The genome of selected individuals from IMCG (n = 6) and DBG (n = 6) parents; previously sequenced by some of the current authors, averaged 6.02-folds coverage which produced raw data of 192.74 Giga base (Gb) then cleaned to reach 191.57 Gb, after filtration and quality check by removing the low-quality and paired reads. The two parents breeds contained 294 and 155 candidate regions, respectively. The top region was located on chromosome 2 of IMCG (113850001:113900001) bp while was located on chromosome 8 (41890001:41950001) bp for DBG. Thus, 176 and 239 selective sweep regions involving 106 and 135 candidate genes were detected in IMCG and DBG respectively^22^. Among them, *FGF-5* was a candidate gene for fibre formation and quality in IMCG based on high genetic differentiation and low heterozygosity. Three synonymous mutations as follows were detected; g. 92103757C>T (rs653636435), g. 92124227A>T (rs665580959) and g. 92111741A>T (rs672199363), and 4 mutations were absent from goat dbSNP-database, namely; g.92124199C>T, g.92124134G>C, g.92124131A>G and g.92124086T>G within the exon sequence of the *FGF-5* gene. Only the candidate mutation (g.92124199G>C) could lead to a ''serine'' to ''leucine'' transition at the 224th position (*p.S224L*)* (Supplementary File 2, Fig. S1) in the amino acid sequence of *FGF-5* gene.

Taking into consideration the differentiation in coat colour (Figs. 1, 6C, 7C) during the genome sequencing of IMCG and DBG, non-synonymous mutation g. 68354798G>A, namely rs647214940, was located on the 16th exon of KIT gene. So, in the current work, this site was examined for the studied goat breeds. The selected animals from the three goat breeds sequenced and examined for *KIT* gene/rs647214940 site (199 bp) revealed the presence of one genotype (AA) for IMCG, three genotypes for DBG (AA = 0.1290), (AG = 0.2903) and (GG = 0.5807), and two genotypes for F_1_ (AA = 0.4118) and (AG = 0.5882), (Table 5 and Fig. 6A,B) and (Supplementary File 1, Fig. 2A,B). The non-synonymous mutation g. 68354798G>A (rs647214940 site) led to ^765^glutamic (Glu)/^765^glycine (Gly) (off white to light brown coat colour) in F_1_, while was ^765^Glu/^765^Glu (white coat colour) in IMCG, and was ^765^Gly/^765^Gly (black coat colour) in DBG (Fig. 6C). Worth mentioning, crossing between IMCG and DBG produced F_1_ progeny having light coat colour (Fig. 7).

**Table 5:** Genotypic and gene frequency of rs647214940 site(*KIT* gene).

Breeds	Coat colour	No	Male	Female	Genotypic frequencies					PIC^4^	*He*^5^
					AA	AG	GG	A	G		
IMCG^1^	White	25	5	20	1	0	0	1	0	0	0
DBG^2^	Black	31	6	25	0.1290	0.2903	0.5807	0.2742	0.7258	0.3980	0.5619
F_1_^3^	White to light brown	17	7	10	0.4118	0.5882	0	0.7059	0.2941	0.4152	0.4844

^1^*IMCG* Mongolia Cashmere goat, ^2^*DBG* Dazu black goat, ^3^*F*_*1*_ IMCG*x*DBG cross, ^4^*PIC* polymorphism information content, ^5^*He* heterozygosity.

## Discussion

Cashmere fibres produced by goats are a luxury raw-material that has been utilized in the textile industry for several centuries. It imparts texture, beauty, softness, brightness, elasticity, durability and colour to the fabrics^16,23,24^. These properties make it conceivable for being a desirable fibre within the textile industry^16^. Cashmere fibres quality characteristics are determined by length, diameter, elasticity, intensity, whiteness and the crimping degree^16,23,24^.

Evaluation of fibre quality characteristics and making comparisons between fibre properties were performed to check if F_1_ cross benefited from combining the inherent cashmere quality and the adaptability of either pure parent. This eventually add to the odds of improving the economics of cashmere production in China.

The hair length obtained on parents breeds was similar to those reported by Guan et al.^22^. F_1_ cross having hair length of 8.90 cm recorded an intermediate value between the pure parents IMCG and DBG, and reflected genotypic heterozygosity (Table 1). Shoulder and leg hair achieved higher length than side-portion and abdomen of F_1_ cross due to differentces in genetic control of fibre growth. The current hair length values were higher than those of Anatolian native goat (6.10 cm)^25^, Krygyzstan (5.00 cm)^16^ Gorno Altai cashmere goat (4.50 cm)^26^ and hair goats in Turkey (4.40 cm)^16^. On the contrary to IMCG and F_1_, hair length values obtained on DBG were humble to those of Chinese hair goats (4.89 cm), Kilis goats (5.30 cm)^27^, but similar to some Chinese hair goats (3.80 cm), and higher than those of Norduz goats (2.44 cm)^28^.

The hair diameter of DBG (92.42 μm) was much higher than that of IMCG (84.60 μm) but F_1_ had a diameter of 86.38 μm, much closer to that of IMCG which indicated that crossing affected hair diameter of F_1_ noticeably towards the finess hair. There is a general agreement that hair diameter is a highly heritable trait. Estimates of heritability of fibre diameter included values of 0.41^29^, 0.51, 0.61 and 0.99^17^, indicating a good opportunity for progress by selection and breeding programs towards production of fine hair. Hair diameters of all body sites of F_1_ were closer to or even higher than that of fine hair of IMCG except leg site which was the lowest but still higher than that of DBG. These results revealed that hair diameters of all studied breeds were thicker than those of hair goat cashmere (14.30 μm)^26^, Turkish hair goats (16.00–18.20 μm), Kilis goats (16.20 μm), Savannah goats (16.00–18.50 μm), Boer goats (16.20–18.50 μm)^26^, Gorno Altai goat (18.50–19.00 μm) and Van-Turkish hair goats (17.32–17.94 µm)^16^.

The current cashmere length values for parents breeds were higher than those of South African domestic goats (1.5–3.0 cm)^26^, Turkish hair goats (2.80 cm)^30^, Van-Turkish hair goats (2.50–2.96 cm)^16^, South African Boer (2.00–3.10 cm), Savannah goats (2.00–3.10 cm)^26^, Anatolian native goat (3.30–6.10 cm)^25^ and Norduz goat (2.44 cm)^28^, while were lower than those of Chinese cashmere (4.89 cm), Kilis goats (5.30 cm)^27^ and Krygyzstan cashmere goat (5.00 cm)^16^. The cashmere length of IMCG (4.55 cm) was similar to that of Gorno Altai cashmere goat (4.50 cm)^26^. On the contrary to IMCG and DBG, the fibre length values obtained on F_1_ were lower than those of Boer goats, Savannah goats (3.10 cm)^26^ and Anatolian goat (3.30 cm)^25^.

As for cashmere diameter, F_1_ possessed a value of 16.27 μm, being the highest while no differences existed between DBG and IMCG (Table 2). These values for parents breeds were similar to those reported by Göktepe et al., 2018 on Anatolian native goat breeds (15.00 µm)^25^, British milk goats (14.00–15.00 μm), cashmere hair goat (14.30 μm), Chinese cashmere goats (14.50–15.50 μm)^16^, (14.63–15.75 μm)^29^ and South-African domestic goats (14.00–16.50 μm). While, the cashmere diameter values obtained on F_1_ were similar to Turkish hair goats (16.00–18.20 μm), Kilis goats (16.20 μm), Savannah goats (16.00–18.50 μm), Boer goats (16.20–18.50 μm)^26^ and Cashmere goats (16.50–17.50 μm)^16,26^, but were lower than those of Gorno Altai goats (18.50–19.00 μm)^16^, Iranian and Afghanistan cashmere goats (17–18.50 μm)^26^ and Van-Turkish hair goats (17.32–17.94 µm)^16^. Worth mentioning, cashmere fibre diameters of more than 16.5 μm is generally utilized in a fabric weave mixing with wool^16,31^. The diameter of the fibre for processed cashmere should be less than 19 μm. A value among 14–19 μm on average is considered appropriate^16^.

On the contrary, another study by Pallotti et al.^32^ measured the effect of age on the length of hair and cashmere, and cashmere diameter in Chinese Alashan Left-Banner White-Cashmere goat, and they reported that all of the above parameters were significantly influenced by the age, but in the current study, the experimental goats from different three breeds were randomly selected to be of the same age (32.50 ± 01.25 months) to measure the effectiveness of the breed (genotype).

Cashmere fibre elasticity measured as curl recovery rate was high for F_1_ (78.07%) compared to IMCG (74.49%) and DBG (77.12%). These values were also higher than those found by Tuncer^16^ for Turkish goats in the Central districts of Van (31.42%), Başkale (25.63%) and Özalp (28.84%) and were also higher than Turkish goat down fibre cashmere (33.00–38.80%)^33^ and Australian cashmere (50%)^34^.

Cashmere fibre strengths of IMCG, DBG and F_1_ being 15.64, 40.07 and 33.99 cN/dT, respectively were lower than those found on Turkish hair goat (45.60–46.60 cN/dT)^16^, Australian cashmere (110.20 cN/dT)^34^ and Anatolian native goat (< 120 cN/dT)^25^. However, DBG and F_1_ cashmere strengths contrary to IMCG were higher than those reported by Mehmet et al. for hair goats (25.00 cN/dT)^33^.

The cashmere whiteness measured colourimetrically as the degree to which the cashmere surface is white. IMCG recorded the highest degree of whiteness (34.98) followed by F_1_ which again was intermediate between parents (31.28) whilst the lowest was obtained on DBG (26.98).

Comparing between breeds, the cashmere yield point of F_1_ (28.98 cN) was very close to that of the high DBG value (32.55 cN) and much higher than that of IMCG parent (11.51 cN). The elongation values for DBG (25.34%) and F_1_ (27.02%) were lower than that for IMCG and that was similarly reported by Göktepe et al.^25^ on Anatolian goat breeds. Surprisingly, sex had no effect on all hair and cashmere properties but this agrees with the results of Mehmet et al. on Turkish hair goat^33^.

The cortical cells appear under the microscope as spindle-shaped and is aligned parallel to the fibre axis (Fig. 5). Generally, cortical cells consist of many types: para, ortho and intermediate mesocortical with differentiation in chemical and physical properties^35^. In this regard, Satlow^36^ made a comprehensive comparison between several morphological investigations and concluded that Mongolian cashmere fibre was bilateral and the amount of para material in the wool was more than in cashmere. On the contrary to the findings of Roberts^37^ cashmere (49.6% para and 50.4% ortho) was different from lambs-wool of similar fibre diameter (34.8% para and 65.2% ortho).

The current cortical cell dimensions of IMCG (94.57 μm) being longer than that of DBG (86.85 μm), while lacking difference in diameters, were different from those of McGregor and Liu who reported that the cashmere goats produced longer (50.8 μm) and thicker (4.57 μm) cortical cells with high levels of nutrition, but shorter (34.5 μm) and thinner (4.14 μm) cells with low levels of nutrition^38^. In accordance to this, goats in the current study were under the same management, nutrition level and conditions, therefore, the values of cortical cell dimensions of IMCG and DBG were not different (*P* > 0.1) but were higher than those reported by McGregor and Liu^38^. McGregor and Quispe^39^ studied the dimensions of fibre cortical cells of Peruvian alpaca (rare animal) and reported the values of (42.50–57.30 μm) and (4.50–4.90 μm) for length and diameter.

Coat colour is one of the most important qualitative traits to distinguish between farm animals^40^. In the present study, IMCG and F_1_ were light coat colour, while DBG had dark coat colour. The dark coat colour of animals forms an adaptive mechanism necessary for the regulation of temperature, especially in the cold seasons. Light coat colour is advantageous under intensive radiation environments because of its reflectance property^41^. The coat colour influences radiant heat loss exerting an effect on the animal weight and some productive traits in different livestock species, especially under tropical environments^18,19^.

The variability in goat coat colour is an indication of the indiscriminate crossing existing between different goat breeds. Also, the variability in the morphological characteristics may reflect an adaptability advantage to different ecological systems under which goats are raised including vegetation features pertaining to those zones^42^. Worth mentioning, the pigmentation of the coat colour is genetically regulated by several genes that control the packaging of pigment granules, the amount, pigment-cell number, and type, also, the transfer of pigments to surrounding-cells^40^.

Several genes have been reported to be correlated with different hair/cashmere properties, in many goat breeds. There are candidate genes related to cashmere fibre properties which were identified in many genomic regions in the selective sweep, involving; *FGF-5*, *KIT*, *OXTR*, *PRKCD*, *IGFBP-7*, *SGK-3*, and *ROCK-1*^43^. Li et al. identified 135 genomic regions that were associated with hair/cashmere properties within the cashmere goat populations. These selected genomic regions contained genes, which are potentially involved in the production of the hair and cashmere fibre, such as *FGF-5*, *IGFBP-7* and *ROCK-1*^43^. Hair/cashmere growth was supposedly regulated by the hairless, hypertrichosis, hair over-growth, *FGF-5*, *FOXI-3*, *Sox-9*, *Trps-1* and *ABCA-5* genes^44^. However, natural long fibre resulted from the regulator *FGF-5* gene, in several species of mammals^44^. Moreover, He et al. reported that the expression of *FGF-5s* and a mRNA alternative splicing of this gene, probably act as the main cause of increasing the cashmere fibre length^45^.

Hair/cashmere length was a characteristic trait in IMCG and its F_1_ cross in comparison to DBG. In a previous study, that was carried out by some of the current authors, on selected individuals of parents breeds^22^, the genome sequence of IMCG breed was found to contain 294 candidate regions, involving 176 selective sweep regions and 106 candidate genes including *FGF-5* which had 3 synonymous mutations while 4 other mutations were absent which may lead to differentiation in the hair/cashmere characteristics, especially length^22^. According to several investigations, *FGF-5* gene has a high effect on hair/cashmere formation and natural hair growth variation^46–49^ (Supplementary File 2, Fig. 1C). Suzuki et al.^50^ reported that *FGF-5* gene can produce a full-length protein (*FGF-5*) and a short form (*FGF5s*) through mRNA alternative splicing. In the current study, this gene is located on goat chromosome 6 (92103281–92124341), with two transcripts (*FGF-5* and *FGF-5S*), *FGF-5* transcript contains 3 exons, while *FGF-5s* contains 2 exons (Supplementary File 2, Fig. 1A). It plays a necessary role in hair length in many species^51^. Editing of *FGF-5* gene in goat embryos resulted in an increased number of secondary hair follicles and longer fibre length which would suggest greater cashmere production^20^. A mutation in *FGF-5* was able to grow secondary hair-follicles and produce longer fibres in Cashmere breeds^43^.

Moreover, coat colour plays an important role in the adaptive mechanism through the regulation of temperature especially in severe cold or extremely hot areas. According to Odubote coat colour influences radiant heat loss exerting an effect on animal economic traits in many species, especially goats in tropical environments^18^. Crossing between IMCG and DBG produced F_1_ progeny having light coat colour because white is dominant over black. The mutation site rs647214940 (*KIT* gene) of IMCG may have descended from the white coat colour of ancient cashmere through mutations. The alleles at this locus are either A or G, and therefore three genotypes: AA, AG or GG may exist. Among them, the dominant allele A is for the white coat, and allele G is the recessive white coat. There is a differentiation in the rs647214940 site (*KIT* gene), where three genotypes are in DBG, but two only in F_1_, while only one was found. This coincides with the difference in coat colour for the three breeds.

In this regard, *KIT* gene plays a key role in different colour patterns in the goat, pig, cat, cow, mouse, horse, rabbit, dog and camel^7^, and was found to be associated with white coat in Holstein cattle^52^. Nazari-Ghadikolaei et al. detecting a significant association with coat colour in mohair goats, reported that *KIT* and *PDGFRA* genes on chromosome 6 were found to be related to light coat colour, while *ASIP*, *AHCY*,* RALY* and *ITCH* genes were found to be associated with dark coat colour on chromosome 13^53^.

On the other hand, Salo et al. and Sansregret et al. confirmed that there are 24 genes, including; *CUX-1* and *PLOD-3* play a necessary role in type and formation of hair/cashmere. The *CUX-1* is associated with wavy hair and curly whiskers in mouse^54^. The *PLOD-3* play a role in the formation of hair or in their texture in Angora and Ankara goat breeds^21^. The keratin (*KRT*) and keratin-associated protein (*KRTAP*) family genes located on chromosomes 1 and 5 were highlighted as candidate genes potentially responsible for diameter and kemp traits in Angora goats and have been previously confirmed in sheep^55,56^. In Rayini goat breed there were several regions on chromosomes 2, 5 and 13 related to Cashmere yield^57^. However, the genes related to fibre properties in the studied goat breeds have not been extensively investigated yet, therefore more investigations are needed in this area.

## Methods

### Animals

Goats used in this investigation were kept under a semi-intensive system, in the Dazu Black farm, Southwest University, Beibei, Chongqing, China (29°48′44″N, 106°24′52″E). Totals of 52192369) ♂, 2850♀), 2130 (1010♂, 1120♀) and 2981 (1396♂, 1585♀) hair/cashmere samples have been collected from individual goat body sites; shoulder (A), side-portion (B), abdomen (C), and leg (D) of DBG (n = 203; ♂99, ♀104), IMCG (n = 65; 21♂, 44♀) and F_1_ (n = 79; 39♂, 40♀) goats, respectively, at a rate of 6–12 samples per site for each animal, to assess the hair/cashmere properties and quality characteristics. The experimental goats from the three breeds were adults (32.50 ± 01.25 months) randomly selected from the respective groups of contemporaries born within 2–5 weeks. All goats were raised under the same conditions of feeding, weather and management. The F_1_ goats were produced from crossing between IMCG does with normal estrus and DBG bucks using artificial mating.

### Traits of concern

The hair fibre traits of concern were hair length and hair diameter, while the cashmere fibre traits were: cashmere length, cashmere diameter and cashmere quality measured as; natural length, straight length, cashmere elasticity; curl degree (J), curl recovery rate (JW/%), curl modulus (JD/%), cashmere intensity; elongation at break (mm), breaking strength (cN), work (cN*mm), strength (cN/dT), EYS1.5 (mm) ''Elongation corresponding to 1.5 times the yielding stress in a stress–strain curve'', yield point (cN), elongation (%), cashmere whiteness, coat colour and finally measurements of cortical cells of the samples. All these traits were measured professionally from the four different body sites of both sexes. The units and definitions used in the measurements are shown in Supplementary File 1, Table S1.

### Fibre length and diameter

The guard hair (outer coating coarse) and down-fibres of cashmere were collected manually from individual goat body sites in spring (April and May) using a fleece comb and electric scissors (Tomhousetrade Trading Co., Ltd and Flyco, FC5905, Shanghai Flyco Electrical Appliance Co., Ltd). Fibre length was measured using a metal ruler, and fibre diameter using (400 ×) digital microscope (Olympus IX51 Inverted Microscope Pred IX53—11629, Shinjuku-ku, Tokyo, Japan) with CellSens Standard software (https://www.news-medical.net/cellSens), according to Peterson and Gherardi^58^. Naturally, spiky and coarse hair of outer-coat (hair) upheld among the undercoat cashmere fibres were removed manually by the dehairing process, to measure both separately.

### Cashmere quality testing

Cashmere quality testing was carried out at Southwest University (GPS; 29.813379, 106.417847) and Gansu Agricultural University (GPS; 36.090339, 103.702168). Electronic single fibre strength tester device ''YG001B'' (http://en.czsgddkj.com/products/926.html) was used to estimate the mechanical properties of fibres, and fibre crimp elasticity tester device ''YG362A'' (https://bonnin-tech.en.alibaba.com) was used to measure other quality traits of the studied goat breeds.

### Coat colour measuring

Data on coat colour have been collected professionally by visual assessment for the three studied breeds. Also, 20 IMCG does with normal estrus were selected for artificial mating with 5 DBG bucks to produce the first cross F_1_ (n = 57; 25♂, 32♀) to examine the coat colour for the progeny.

### The measurements of cortical cells

Cortical cells were measured according to the following steps; (1) few fibres of each sample were placed on a glass slide. (2) A drop of concentrated (95%) sulphuric acid has been dripped on the sample and covered with a coverslip. (3) After 2–3 min, the coverslips were pressed and ground gently with tweezers. (4) Cortical cells were, then isolated and observed under the digital microscope (400 ×) with CellSens Standard software. (5) The CellSens Standard software was used for real-time observation and measurement of the length and diameter of the cortical cells. This test was done only on the pure breeds (IMCG and DBG parents).

### DNA isolation, amplification, sequencing and genotyping for Kit gene

Total genomic DNA was isolated from blood samples of randomly selected IMCG (n = 25; 5♂, 20♀), DBG (n = 31; 6♂, 25♀) and F_1_ (n = 17; 7♂, 10♀) experimental goats with DNA isolation-kit (Tiangen Biotech, Beijing, China). The DNA samples were separated by electrophoresis on 1.0–1.2% agarose in 0.5 × TBE buffer according to Sambrook et al.^59^ which contained 0.5 μg/ml ethidium-bromide. The electrophoresis run was performed using apparatus with power-supply and visualized by ultraviolet transilluminator and Gel-documentation system (Chemi.Doc™ XRS + with Image Lab™ Software, BIO-RAD, USA). The purity and integrity of DNA were appropriate, the OD260/280 was 1.82, and the average values of degree, volume and mass were 70.47 ng/μL, 29.5 μL and 2.14 ng, respectively.

The specificity of the PCR primer targeting *KIT* gene (199 bp fragment from exon 16) was carried out using Primer Premier 5.0 software (https://macdownload.informer.com/advice/Primer_Premier_5.html), as follows; F: 5′-TGCCTGCAAGT TCACATCAG-3′ and R: 5′-AAAGCTCAGCAAGTCCTCCA-3′, that synthesized by (Shanghai-Sangon Biolo. Engin. Tech. & Ser. Co., Ltd). The amplification was performed using (Green-Super.mix, TaKaRa, Japan), ten p.mol of the primer and 80–100 ng of each sample of genomic DNA were processed under the following PCR amplification conditions; 34 cycles of denaturation (94 °C/1 min), annealing (57.8 °C/1 min), extension (72 °C/1 min) and a final extension step at 72 °C/2 min. The amplification was carried out using a Thermo-cycler T100 (621BR07645, Singapore).

The sequence analysis was carried out for *KIT* gene by DNA Sequencer (DNBSEQ-G400, GMI, Shanghai, China). The resulted sequences were analyzed using ExPASy program (http://web.expasy.org/translate) and MEGA 6.0 V4. The genetic indices of the three studied populations; polymorphism information-content (PIC) and gene-heterozygosity (*He*) were calculated according to Nei’s methods^60,61^.

### Statistical analysis

All data were tested for normality with the Shapiro–Wilk test using PROC-UNIVARIATE procedure of SAS 9.0 (SAS, 2009) and results indicated that all data were distributed normally. Also, Bartlett's test^62^ has been used for testing the homogeneity of variance for all studied factors for each trait and the results were not significant at all. The same results have been obtained by Levene's Test^63^ which is widely considered to be the standard homogeneity of variance test.

The dependent variables were tested using the GLM procedure of SAS 9.0 (SAS, 2009) according to the following model:

1 $${\text{Y}}_{{{\text{ijk}}}} = \upmu + {\text{B}}_{{\text{i}}} + {\text{S}}_{{\text{j}}} + {\text{BS}}_{{{\text{ij}}}} + {\text{e}}_{{{\text{ijk}}}}$$

where Y_ij_ is an observation on each studied trait, μ is the mean, and B_i_ is the fixed effect of ith (i = 1:3) breed, S_j_ is the fixed effect of the jth sex (j = 1:2), BS_ij_ is the interaction between breed and sex and e_ijk_ is the residual error. Significances among means were tested using least significant differences (LSD, *P* < 0.05).

### Approval for animal experiments: ethics approval

All animals and sampling procedures in this experiment were supervised and approved by the institutional Animal Care and Use Committee of Southwest University, College of Animal Science and Technology, China (2019, No. GB14925-2010). All procedures and experimental protocols were in accordance with the Guide for the Care and Use of Agricultural Animals in Research and Teaching, Federation of Animal Science Societies (FASS, 2010) https://www.aaalac.org/about/Ag_Guide_3rd_ed.pdf. Also, the study was carried out in compliance with the ARRIVE guidelines (https://arriveguidelines.org).

## Conclusion

Superior properties of cashmere fibres made it known as “fibres of kings”, presenting a valuable high-quality source for textile products in China. In this respect, the importance, characteristics and genetic background of the cashmere fibres especially ''down-fibres'' of IMCG and its first cross was highlighted in the present work. The characteristics of F_1_ hair/cashmere were analysed and reported for the first time. The present results indicated that high hair length was obtained for IMCG followed by F_1_ with significant difference from DBG, on contrary to hair diameter. IMCG and F_1_ recorded the highest of most cashmere quality traits. *FGF-5* was detected as a candidate gene in IMCG, with significant effects on several of hair/cashmere traits. While, *KIT* gene was found to be associated with coat colour. More trials to detect hair/cashmere genes in different breeds and test their effects on hair/cashmere production and benefits to the industry are necessary.

## Supplementary Information

Supplementary Information 1.

Supplementary Information 2.

## Data Availability

All data generated or analyzed during this study are included in this manuscript and its supplementary information files.
