# Supplementary material for: Assessment of hair and cashmere properties and their genetic background of several goat breeds in Southwest China
Source: Sci Rep. 2022 Jul 1;12:11135. doi: 10.1038/s41598-022-14441-1 (PMC9249783; doi:10.1038/s41598-022-14441-1)
Supplement: Supplementary file 1 — Supplementary Information 1. [file 41598_2022_14441_MOESM1_ESM.pdf]

Supplementary File 1

Table S1. The units and definitions which used in the measurements.

Traits	Definition	Unit
**Length**	The length of fibre is defined as "the length of test specimen that taken by a metal ruler", in the current study**.**	cm (centimeter)
**Diameter**	The diameter of the fibre is defined as "the diameter of test specimen that taken by digital microscope with CellSens Standard software", in the current study.	µm (micro-metre)
**Curl degree**		J
**Curl recovery rate**		JW/%
**Curl modulus**	Modulus is the property of a material representative of its resistance to deformation.	JD/%
**Elongation at Break**	*****It is the ratio between increased length and initial length after breakage of the tested specimen at a controlled temperature.<br><br>***** Elongation at Break (fracture strain / tensile elongation at break), is the ratio between increased length and initial length after breakage of the tested specimen at a controlled temperature. It is related to the ability of the sample specimen to resist changes of shape without cracking.	mm (Millimeter)
**Breaking strength**	*****The average of the results of breaking force measurements.	cN (city inch "China")
**Work**		cN*mm<br>(city inch)*Millimeter)
**Strength (Tenacity)**	*****The tensile strength expressed as force per unit liner density is called tenacity. This is normally expressed as gram force per tex (gf/tex).	cN/dT<br>(city inch"China"/ decitex)<br>1 Ktex = 1,000 tex<br>1 decitex = 1/10 tex<br>1 militex = 1/1,000 tex
**EYS1.5**	*****Elongation corresponding to 1.5 times the yielding stress in a stress-strain curve.	mm (Millimeter)
**Yield point**		cN (city inch "China")
**Elongation %**	*****Is the measure of the extent of deformation along the axis of the fibre under tensile stress expressed as a percentage change in length based on the original length of test sample.<br>*****The percentage of length up to which the yarn can be extended before the occurrence of yarn breakage.<br>*****It is the elasticity of the yarn in %, e.g. 14.90 % = you can stretch the yarn to 14.90 % until it breaks.<br>*****The higher the breaking energy = better running characteristics, in general, a higher elongation is better for knitting and yarn quality.	% (Percentage)

Supplementary File 1

Table S2. The comparison of different genders and sites for hair length and diameter of IMCG[1], DBG[2] and their first cross F_1[3] (Means).

Hair traits	Breed								
	IMCG[1]			DBG[2]			F_1[3]		
**Length (cm)**									
**Gender**	♂	♀	**Overall mean**	♂	♀	**Overall mean**	♂	♀	**Overall mean**
Shoulder	18.562	17.210	18.380[a]	4.230	4.182	4.032[c]	11.568	11.541	11.406[b]
Side-portion	11.091	11.622	11.975[a]	4.108	3.945	4.015[c]	6.692	6.561	6.797[b]
Abdomen	9.603	9. 473	9.955[a]	4.509	3.916	4.120[c]	6.351	6.233	6.350[b]
Leg	16.680	16.051	15.630[a]	4.486	4.390	4.400[c]	11.220	10.901	11.019[b]
**Diameter (µm)**									
**Gender**	♂	♀	**Overall mean**	♂	♀	**Overall mean**	♂	♀	**Overall mean**
Shoulder	85.370	89.448	88.910[c]	90.100	9.230	91.044[a]	89.852	90.347	90.495[b]
Side-portion	79.164	84.325	84.182[b]	97.120	98.780	99.765[a]	87.330	83.221	83.270[c]
Abdomen	85.135	87.342	85.553[b]	86.642	89.824	89.213[a]	67.334	69.162	68.700[c]
Leg	78.160	82.911	79.773[c]	82.320	88.913	89.678[b]	98.690	104.313	103.061[a]

[1] **IMCG**; Mongolia Cashmere goat, [2] **DBG**; Dazu black goat, [3] **F_1**; IMCG X DBG cross.
[a-c] Means with different superscript in the same row are differ (P<0.05).

Supplementary File 1

Table S3. The comparison of different genders and sites for cashmere length and diameter of IMCG[1], DBG[2] and their first cross F_1[3] (Means).

Cashmere traits	Breed								
	IMCG[1]			DBG[2]			F_1[3]		
**Length (cm)**									
**Gender**	♂	♀	**Overall mean**	♂	♀	**Overall mean**	♂	♀	**Overall mean**
Shoulder	5.332	5.040	5.012[a]	3.821	3.672	3.679[b]	2.305	2.563	2.411[c]
Side-portion	4.465	4.124	4.243[a]	3.530	3.633	3.061[b]	2.377	2.206	2.223[c]
Abdomen	4.521	4.221	4.512[a]	2.340	2.482	2.336[b]	2.105	2.302	2.209[b]
Leg	5.301	5.012	5.003[a]	2.360	2.821	2.407[b]	2.294	2.247	2.347[b]
**Diameter (μm)**									
**Gender**	♂	♀	**Overall mean**	♂	♀	**Overall mean**	♂	♀	**Overall mean**
Shoulder	13.392	12.558	14.929[b]	14.393	15.670	15.147[ab]	16.811	17.021	16.904[a]
Side-portion	13.524	12.066	13.417[c]	15.020	15.872	15.187[a]	15.233	15.653	15.409[a]
Abdomen	13.351	13.170	13.327[c]	14.705	14.896	14.969[a]	14.942	15.563	15.348[a]
Leg	14.682	14.890	14.468[b]	14.890	15.100	15.024[ab]	15.528	16.883	16.913[a]

[1] **IMCG**; Mongolia Cashmere goat, [2] **DBG**; Dazu black goat, [3] **F_1**; IMCG*x*DBG cross.
[a-c] Means with different superscript in the same row are differ (P<0.05).

Supplementary File 1

Table S4. The comparison of different genders and sites for cortical cell length and diameter in parents goat breeds; IMCG[1] and DBG[2] (Means).

**Cortical cells**	**Breeds**					
	**IMCG[1]**			**DBG[2]**		
**Length (µm)**						
Gender	♂	♀	**Overall mean**	♂	♀	**Overall mean**
Shoulder	93.011	91.515	91.565[a]	89.362	85.200	86.908[b]
Side-portion	89.091	93.592	93.933[a]	84.910	91.703	86.788[b]
Abdomen	98.253	94.757	97.150[a]	86.955	85.883	86.828[b]
Leg	100.010	89.416	94.215[a]	89.201	86.564	86.840[b]
**Diameter (µm)**						
Gender	♂	♀	**Overall mean**	♂	♀	**Overall mean**
Shoulder	4.979	5.520	5.588	4.981	5.322	5.183
Side-portion	5.371	5.922	5.928	5.250	5.651	5.473
Abdomen	6.166	6.982	6.870[a]	5.353	5.418	5.413[b]
Leg	4.753	5.914	5.860	5.334	5.386	5.355

[1] **IMCG**; Mongolia Cashmere goat, [2] **DBG**; Dazu black goat.
[a-b] Means with different superscript in the same row are differ (*P*<0.05).

Supplementary File 1

Table S5. The comparison of different genders for natural and straight lengths of cashmere of IMCG[1], DBG[2] and their first cross F_1[3] (Means).

Quality traits	Breeds		
**Cashmere Length (cm)**	**IMCG[1]**	**DBG[2]**	**F_1[3]**
**Natural length**			
♂	4.422	3.013	2.466
♀	4.552	3.152	2.223
**Overall mean**	4.549[a]	3.082[b]	2.352[c]
**Straight length**			
♂	5.606	3.501	3.345
♀	6.010	3.914	4.012
**Overall mean**	5.896[a]	3.869[c]	3.916[b]

[1] **IMCG**; Mongolia Cashmere goat, [2] **DBG**; Dazu black goat, [3] **F_1**; IMCG X DBG cross.
[a-c] Means with different superscript in the same row are differ (P<0.05).

Supplementary File 1

Table S6. The comparison of different genders for cashmere elasticity of IMCG[1], DBG[2] and their first cross F_1[3] (Means).

Quality traits	Breeds		
**Cashmere elasticity**	**IMCG[1]**	**DBG[2]**	**F_1[3]**
**Curl degree (J)**			
♂	2.901	3.600	2.980
♀	2.570	3.452	2.790
**Overall mean**	2.862[b]	3.574[a]	2.890[b]
**Curl recovery rate (JW/%)**			
♂	75.323	78.101	78.450
♀	74.341	76.904	77.120
**Overall mean**	74.490[b]	77.121[a]	78.071[a]
**Curl modulus (JD/%)**			
♂	2.100	2.344	1.781
♀	2.223	2.132	1.985
**Overall mean**	2.138[b]	2.298[a]	1.912[c]

[1] **IMCG**; Mongolia Cashmere goat, [2] **DBG**; Dazu black goat, [3] **F_1**; IMCG X DBG cross.
[a-c] Means with different superscript in the same row are differ (P<0.05).

Supplementary File 1

Table S7. The comparison of different genders for cashmere intensity of IMCG[1], DBG[2] and their first cross F_1[3] (Means).

Quality traits	Breeds		
**Cashmere intensity**	**IMCG[1]**	**DBG[2]**	**F_1[3]**
	**Elongation at break (mm)**		
♂	4.240	2.811	2.330
♀	5.004	2.987	2.677
Overall mean	4.955[a]	2.956[b]	2.593[b]
	**Breaking strength (cN)**		
♂	12.550	38.379	28.003
♀	12.121	37.998	26.945
Overall mean	12.266[c]	38.287[a]	27.91[b]
	**Work (cN*mm)**		
♂	41.325	60.011	47.110
♀	39.875	59.133	46.650
Overall mean	40.332[c]	59.536[a]	46.983[b]
	**Strength (cN/dT)**		
♂	15.012	39.670	34.120
♀	16.134	41.230	32.998
Overall mean	15.641[c]	40.071[a]	33.990[b]
	**EYS1.5 (mm)**		
♂	3.986	2.234	2.103
♀	4.127	2.301	1.980
Overall mean	4.019[a]	2.215[b]	1.990[b]
	**Yield point (cN)**		
♂	10.816	31.326	27.110
♀	11.660	33.126	28.992
Overall mean	11.519[c]	32.555[a]	28.982[b]
	**Elongation (%)**		
♂	47.340	24.670	28.012
♀	50.133	25.780	27.590
Overall mean	49.562[a]	25.347[c]	27.022[b]

[1] **IMCG**; Mongolia Cashmere goat, [2] **DBG**; Dazu black goat, [3] **F_1**; IMCG X DBG cross.
[a-c] Means with different superscript in the same row are differ (P<0.05).

Supplementary File 1

Table S8. The comparison of different genders and sites for cashmere whiteness of IMCG[1], DBG[2] and their first cross F_1[3] (Means).

Quality traits	Breeds								
**Cashmere Whiteness**	**IMCG**[1]			**DBG**[2]			**F_1**[3]		
**Site/Gender**	♂	♀	Overall mean	♂	♀	Overall mean	♂	♀	Overall mean
Shoulder	34.912	35.012	35.098	25.400	27.570	27.514	29.980	31.430	31.173
Side-portion	32.915	35.675	35.519	26.780	27.630	27.324	32.222	32.100	32.175
Abdomen	34.130	36.130	35.506	25.120	27.023	26.546	30.120	31.331	31.050
Leg	32.901	33.534	33.781[a]	26.133	26.034	26.534[c]	28.950	30.845	30.750[b]

[1] **IMCG**; Mongolia Cashmere goat, [2] **DBG**; Dazu black goat, [3] **F_1**; IMCG X DBG cross.
[a-c] Means with different superscript in the same row are differ (P<0.05).

Supplementary File 1

Table S9. The comparison of different sites for natural and straight lengths of cashmere of IMCG[1], DBG[2] and their first cross F_1[3] (Means).

Quality traits	Breeds		
**Cashmere Length (cm)**	**IMCG[1]**	**DBG[2]**	**F_1[3]**
	**Natural length**		
Shoulder	4.521	4.167	2.363
Side-portion	5.049	4.087	2.045
Abdomen	4.077	3.713	2.043
Leg	4.543	4.188	2.267
**Overall mean**	4.549[a]	4.049[b]	2.352[c]
	**Straight length**		
Shoulder	5.871	4.912	3.967
Side-portion	5.412	4.322	3.256
Abdomen	5.214	4.910	2.924
Leg	6.407	4.549	3.680
**Overall mean**	5.896[a]	4.869[b]	3.916[c]

[1] **IMCG**; Mongolia Cashmere goat, [2] **DBG**; Dazu black goat, [3] **F_1**; IMCG X DBG cross.
[a-c] Means with different superscript in the same row are differ (P<0.05).

Supplementary File 1

Table S10. The comparison of different sites for cashmere elasticity of IMCG[1], DBG[2] and their first cross F_1[3] (Means).

**Quality traits**	**Breeds**		
**Cashmere elasticity:**	**IMCG**[1]	**DBG**[2]	**F_1**[3]
	**Curl degree (J)**		
Shoulder	2.152	2.783	2.230
Side portion	2.133	3.223	2.042
Abdomen	2.518	3.176	2.422
Leg	3.917	4.942	3.170
**Overall mean**	2.862[b]	3.574[a]	2.890[b]
	**Curl recovery rate (JW/%)**		
Shoulder	75.702	80.237	81.113
Side portion	73.106	77.033	79.120
Abdomen	75.263	78.081	78.404
Leg	72.506	76.086	78.016
**Overall mean**	74.49[b]	77.121[a]	78.071[a]
	**Curl modulus (JD/%)**		
Shoulder	1.769	2.282	1.623
Side portion	2.276	2.123	1.457
Abdomen	1.893	2.258	1.723
Leg	2.752	2.367	1.844
**Overall mean**	2.138[b]	2.298[a]	1.912[c]

[1] **IMCG**; Mongolia Cashmere goat, [2] **DBG**; Dazu black goat, [3] **F_1**; IMCG X DBG cross.
[a-c] Means with different superscript in the same row are differ (P<0.05).

Supplementary File 1

Table S11. The comparison of different sites for cashmere intensity of IMCG[1], DBG[2] and their first cross F_1[3] (Means).

Quality traits	Breeds		
	IMCG[1]	DBG[2]	F_1[3]
**Elongation at break (mm)**			
Shoulder	5.071	2.563	2.347
Side portion	4.095	2.756	2.486
Abdomen	4.807	2.244	2.230
Leg	4.987	2.965	2.492
Overall mean	4.955[a]	2.956[b]	2.593[b]
**Breaking strength (cN)**			
Shoulder	8.607	33.651	23.547
Side portion	11.788	37.912	25.017
Abdomen	14.948	41.075	27.564
Leg	13.243	41.844	28.345
Overall mean	12.266[c]	38.287[a]	27.910[b]
**Work (cN*mm)**			
Shoulder	39.448	48.258	45.124
Side portion	40.133	51.353	46.211
Abdomen	44.244	69.861	49.514
Leg	41.304	73.875	47.127
Overall mean	40.332[c]	59.536[a]	46.983[b]
**Strength (cN/dT)**			
Shoulder	13.991	37.730	31.653
Side portion	14.985	40.367	32.190
Abdomen	15.118	40.158	32.781
Leg	16.816	41.334	34.400
Overall mean	15.641[c]	40.071[a]	33.990[b]
**EYS1.5 (mm)**			
Shoulder	4.214	2.422	1.350
Side portion	4.122	2.120	1.542
Abdomen	3.846	2.007	1.890
Leg	3.997	2.431	2.121
Overall mean	4.019[a]	2.215[b]	1.990[b]

[1] **IMCG**; Mongolia Cashmere goat, [2] **DBG**; Dazu black goat, [3] **F_1**; IMCG X DBG cross.
[a-c] Means with different superscript in the same row are differ (P<0.05).

Supplementary File 1

Continued Table S11. The comparison of different sites for cashmere intensity of IMCG[1], DBG[2] and their first cross F_1[3] (Means).

Quality traits	Breeds		
	**IMCG[1]**	**DBG[2]**	**F_1[3]**
**Yield point (cN)**			
Shoulder	10.952	31.526	29.103
Side portion	10.235	30.466	27.890
Abdomen	13.559	30.278	27.616
Leg	12.047	35.884	28.130
Overall mean	11.519[c]	32.555[a]	28.982[b]
**Elongation (%)**			
Shoulder	50.707	23.310	25.022
Side portion	47.934	25.126	25.133
Abdomen	48.074	23.823	26.950
Leg	49.905	27.709	27.012
Overall mean	49.562[a]	25.347[c]	27.022[b]

[1] **IMCG**; Mongolia Cashmere goat, [2] **DBG**; Dazu black goat, [3] **F_1**; IMCG X DBG cross.
[4]SEM; the standard error of the mean.

[a-c] Means with different superscript in the same row are differ (P<0.05).
