# Supplementary material for: Assessment of hair and cashmere properties and their genetic background of several goat breeds in Southwest China
Source: Sci Rep. 2022 Jul 1;12:11135. doi: 10.1038/s41598-022-14441-1 (PMC9249783; doi:10.1038/s41598-022-14441-1)
Supplement: Supplementary file 2 — Supplementary Information 2. [file 41598_2022_14441_MOESM2_ESM.pdf]

Supplementary File 2

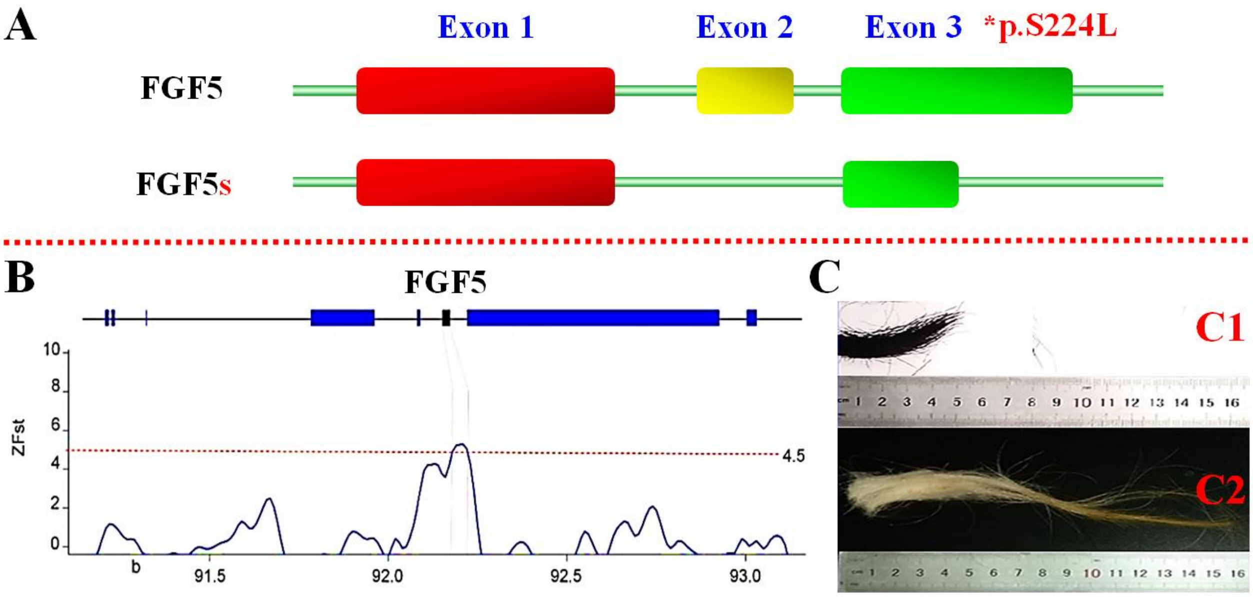

Figure S1. Schematic diagram of FGF5 gene; **(A)** FGF5 gene as a full-length form (FGF5) and a short form (FGF5s). **(B)** *ZFst* value for FGF5 gene. **(C)** The hair length for **C1**: **DBG**; Dazu black goat and **C2**: **IMCG**; Inner Mongolia Cashmere goat.

Supplementary File 2

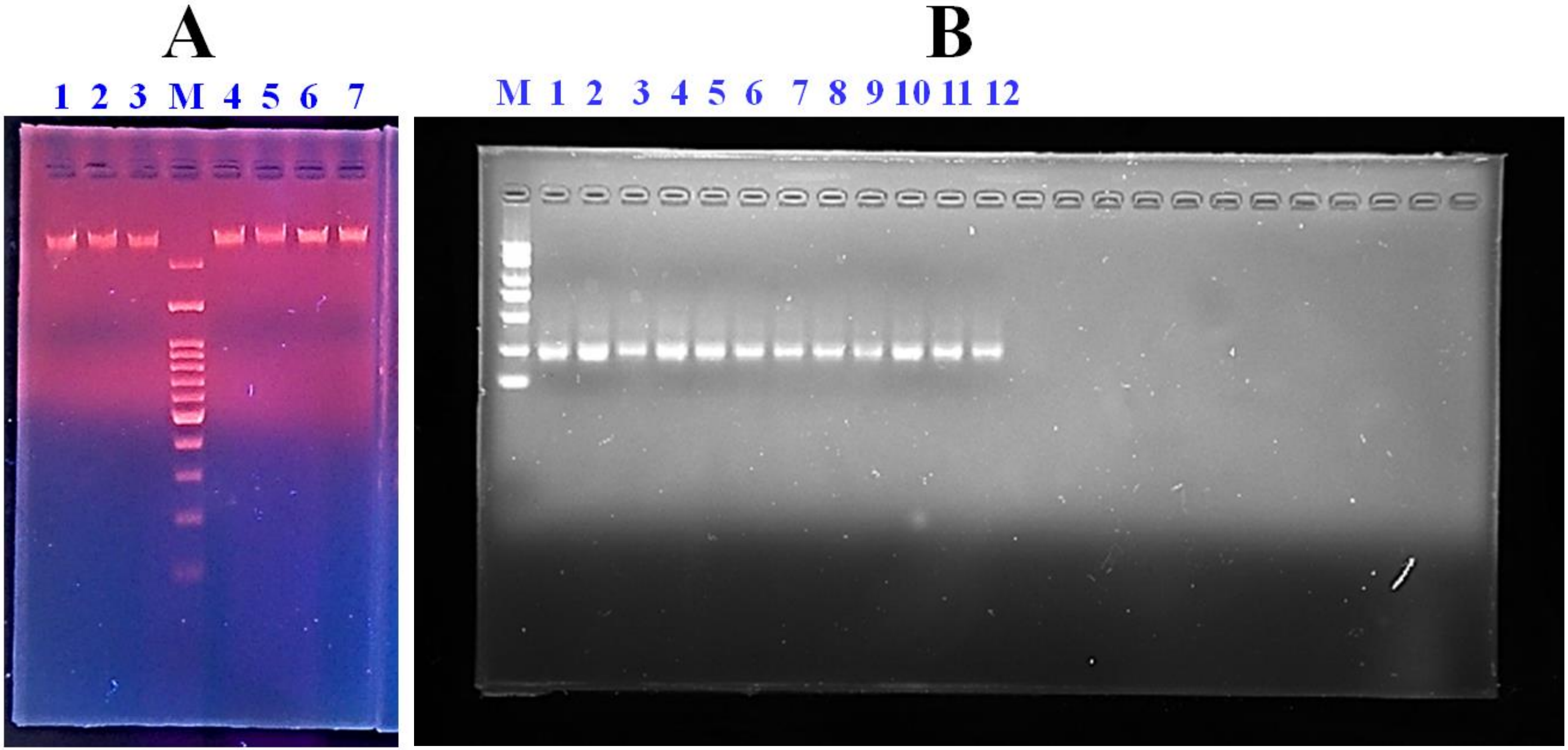

Figure S2. **(A)** Genomic DNA extraction from the blood of experimental goats, Lanes:1 to 3 for Inner Mongolia cashmere goat (IMCG) 1μL/ sample, Lanes: 4 to 5 for Dazu black goat (DBG) 1μL/ sample and Lanes: 6 to 7 for IMCG*x*DBG cross (F_1), M: 100 bp DNA ladder. **(B)** PCR amplification (electrophoretogram) of Kit gene (rs647214940 site), Lanes: 1 to 4 for Inner Mongolia cashmere (IMCG), lanes: 5 to 8 for Dazu black goat (DBG), and lanes: 9 to 12 for IMCG*x*DBG cross (F_1), M: 100 bp DNA ladder.
